# A role for actomyosin contractility in Notch signaling

**DOI:** 10.1186/s12915-019-0625-9

**Published:** 2019-02-11

**Authors:** Ginger L. Hunter, Li He, Norbert Perrimon, Guillaume Charras, Edward Giniger, Buzz Baum

**Affiliations:** 1 National Institute of Neurological Disorders and Stroke, NIH, Bethesda, MD 20892 USA; 2 MRC-LMCB, University College London, London, WC1E6BT UK; 3 Institute for the Physics of Living Systems, University College London, London, WC1E6BT UK; 4 Department of Genetics, Harvard Medical School, Howard Hughes Medical Institute, Boston, MA 02115 USA; 5 London Centre for Nanotechnology, University College London, London, WC1E6BT UK; 6 Department of Cell and Developmental Biology, University College London, London, WC1E6BT UK; 7 Present Address: Department of Biology, Clarkson University, Potsdam, NY 13699 USA

## Abstract

**Background:**

Notch-Delta signaling functions across a wide array of animal systems to break symmetry in a sheet of undifferentiated cells and generate cells with different fates, a process known as lateral inhibition. Unlike many other signaling systems, however, since both the ligand and receptor are transmembrane proteins, the activation of Notch by Delta depends strictly on cell-cell contact. Furthermore, the binding of the ligand to the receptor may not be sufficient to induce signaling, since recent work in cell culture suggests that ligand-induced Notch signaling also requires a mechanical pulling force. This tension exposes a cleavage site in Notch that, when cut, activates signaling. Although it is not known if mechanical tension contributes to signaling in vivo, others have suggested that this is how endocytosis of the receptor-ligand complex contributes to the cleavage and activation of Notch. In a similar way, since Notch-mediated lateral inhibition at a distance in the dorsal thorax of the pupal fly is mediated via actin-rich protrusions, it is possible that cytoskeletal forces generated by networks of filamentous actin and non-muscle myosin during cycles of protrusion extension and retraction also contribute to Notch signaling.

**Results:**

To test this hypothesis, we carried out a detailed analysis of the role of myosin II-dependent tension in Notch signaling in the developing fly and in cell culture. Using dynamic fluorescence-based reporters of Notch, we found that myosin II is important for signaling in signal sending and receiving cells in both systems—as expected if myosin II-dependent tension across the Notch-Delta complex contributes to Notch activation. While myosin II was found to contribute most to signaling at a distance, it was also required for maximal signaling between adjacent cells that share lateral contacts and for signaling between cells in culture.

**Conclusions:**

Together these results reveal a previously unappreciated role for non-muscle myosin II contractility in Notch signaling, providing further support for the idea that force contributes to the cleavage and activation of Notch in the context of ligand-dependent signaling, and a new paradigm for actomyosin-based mechanosensation.

**Electronic supplementary material:**

The online version of this article (10.1186/s12915-019-0625-9) contains supplementary material, which is available to authorized users.

## Background

The actin cytoskeleton plays a major role in the regulation of cell shape and tissue organization in animals [1], and as such is tightly regulated in space and time downstream of many gene regulatory networks and signaling cascades [2, 3]. Conversely, recent work has shown that actin-based protrusions and the forces generated in the actomyosin cortex can also influence the ability of cells to send and receive signals from their environment [4, 5]. This realization spawned the field of mechanotransduction [6]. In considering how mechanical forces might influence cell behavior, it has been proposed that the application of force to a transmembrane protein complex that is coupled to the cytoskeleton leads to a tension-dependent conformational change. This exposes hidden sites in one or more mechanosensitive proteins that are then read in order to initiate a signal [7].

While few signaling systems other than cell-cell and cell-ECM contacts have been shown to be force sensitive, recent work has implicated mechanical force in the activation of Notch receptor [7–12]. In cell culture, pulling forces of between 3.5 and 5.5 pN are required to expose the S2 site of Notch required for cleavage and Notch activation. Importantly, although it is not yet possible to accurately determine levels of tension experienced by Notch-Delta complexes in vivo, this phenomenon is thought to explain the requirement for actin-mediated endocytosis in Notch signaling, whereby Delta endocytosis generates sufficient force to induce Notch cleavage and activation [13, 14]. However, it is also clear that the actomyosin cytoskeleton itself—whether through the activity of protrusions, at the cell cortex, or elsewhere in the cell—generates contractile forces that could influence signaling. In fact, it is hard to understand how endocytosis could generate the force necessary to unfold Notch in the absence of a rigid or contractile actomyosin cortex upon which to pull and/or push against. These considerations raise the question of whether there are instances in which forces generated by the actomyosin cytoskeleton contribute to the activation of Notch [15–17]. As a specific system in which to test this general idea, we used the fly notum [18–20], taking of advantage of the fact that epithelial cells in this tissue engage in local Notch-Delta signaling via lateral cell-cell contacts and signaling at a distance, which is mediated by long actin-rich protrusions. Since Notch signaling influences the decision of cells to assume a bristle or epithelial fate, this process of lateral inhibition leads to the emergence of a pattern of well-spaced bristles.

In the *Drosophila* pupal notum, the activation of Notch is induced via contact with a neighboring cell expressing Delta. This represses the pro-neural fate so that epithelial cells in which Notch is activated tend to remain epithelial in character [18, 20]. Conversely, the maintenance of low levels of Notch activation during this window of development allows the expression of pro-neural genes, which drive cells towards a mechanosensory bristle fate. Here, we investigate the role of actomyosin contractility in Notch signaling during this process using a combination of quantitative live cell imaging and genetic manipulations. By genetically and pharmacologically modulating myosin II activity in vivo, we demonstrate the presence of actomyosin-based forces between basal cellular protrusions in an epithelium. At the same time, we show that a robust Notch response requires myosin II-mediated contractility in both signal sending and receiving cells in vivo and in a cell culture model of Notch-Delta signaling. These data show that decreased myosin II activity is associated with defects in Notch-dependent bristle spacing, making clear the importance of actomyosin-based forces in tissue patterning.

## Results

### Myosin II activity is required for robust Notch signaling

**Fig. 1:**
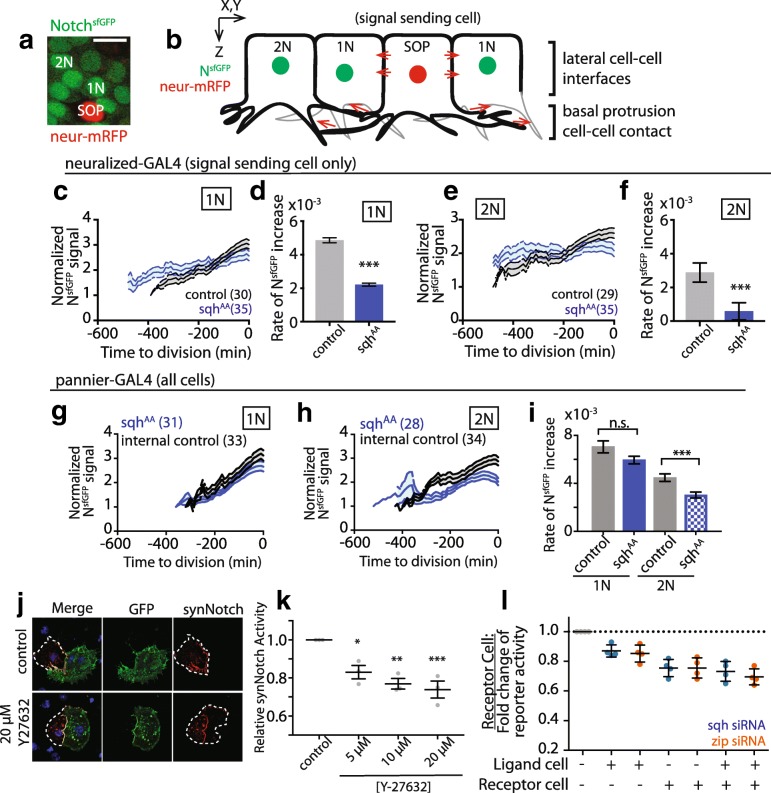
Myosin II activity modulates the Notch response in notum epithelial cells. (**a**) The Notch reporter N^sfGFP^ is visible in epithelial cell neighbors adjacent to SOP (1N) and in epithelial cell neighbors at least one cell diameter away from any SOP cell (2N). Neur-mRFP (neuralized H2B^mRFP^) is expressed to label SOP cell nucleus, scale bar = 10 μm. (**b**) Cartoon model of adjacent Notch signaling via lateral cell-cell contacts and protrusions (1 N) vs cells signaling via basal protrusion contacts alone (2 N). (**c**–**f**) Notch response (mean ± SEM) in wild-type cells (**c**) adjacent or (**e**) distant to SOP cells expressing UAS-spaghetti squash^AA^ (sqh^AA^; blue) or UAS-LifeAct^Ruby^ (black) under the neur-GAL4 driver. (**d**, **f**) Mean ± SEM linear regression slopes for data averaged in (**c**, **e**). ***, *p* ≤ 0.001 by unpaired *t* test. Rate (*y*-axis) represents change in NsfGFP fluorescence per unit time (min). (**g**–**i**) Notch response in tissue where both SOP and epithelial cells express UAS-spaghetti squash^AA^ under the pnr-GAL4 driver. Internal controls were measured outside the pnr domain. Expression of spaghetti squash^AA^ does not affect N^sfGFP^ levels (mean ± SEM) in (**g**) adjacent but does decrease N^sfGFP^ in (**h**) distant cells compared to internal controls, and only decreases the rate of Notch response in distant cells (**i**, mean ± SEM). ***, *p* ≤ 0.001; ns, not significant by unpaired *t* test. (*n*) = total number of nuclei measured, *N* = 3 nota analyzed per genotype. (**j**) Images of heterogeneous cell culture to measure synthetic Notch response to myosin II activity. Cells in green express ligand, those in red express receptor. (**k**) Relative levels of synNotch activation (detected via luciferase assay) in response to increasing concentrations of Y-27632. Mean ± SEM, *p ≤ 0.05, **p ≤ 0.01, ***p ≤ 0.001, by ANOVA with multiple comparisons. (**l**) Relative levels of synNotch activation (detected via luciferase assay) in response to siRNA against zipper (orange) or spaghetti squash (blue), in the presence of DMSO. Plus sign indicates transfection with siRNA; minus sign indicates transfection with control siRNA targeting the white gene. Mean ± SEM for 4 experimental repeats shown. See also Additional file 1: Figure S1

Myosin II motors contribute to the generation of actin-dependent pulling forces to drive a wide range of developmental processes [21–23]. In order to determine whether actomyosin contractility is required for lateral inhibition signaling during notum pattern formation, we asked how decreasing actomyosin tension affects the activity of a transcriptional reporter of Notch signaling, N^sfGFP^ (Fig. 1a, b) [24]. We measured the average accumulation of GFP over time as a reporter of Notch activity (hereafter, rate of Notch response; see the “Methods” section for more detail). We then used the GAL4/UAS expression system to perturb the function of non-muscle myosin II in this background. Non-muscle myosin II is a multimeric motor protein complex whose heavy chain is encoded by the Drosophila gene *zipper*, and regulatory light chain (RLC) is encoded by *spaghetti squash* [25, 26]. Previous work showed that loss of function mutations and/or expression of dominant negative derivatives of *zipper* or RLC leads to phenotypes consistent with decreased cortical tension [22, 27]. Since animals homozygous mutant for null alleles of *zipper* (or *spaghetti squash)* are not viable to pupariation, we used tissue-specific expression of constructs designed to perturb myosin II function in specific populations of cells to assess the impact of myosin II on Notch signaling in the notum. These include Zipper^DN^, a motor-less heavy chain protein that binds and sequesters wild-type heavy chain, thus lowering contractility [22], a non-phosphorylatable variant of the RLC, spaghetti squash^AA^ [27], or RNAi-mediated silencing of Rho kinase (ROK), an upstream activator of myosin II contractility [28]. In our experiments, we find that these constructs are associated with phenotypes of varying severity. The expression of Zipper^DN^ was associated with the strongest phenotypes, followed by spaghetti squash^AA^, while the expression of RNAi constructs had the least severe effect. This is consistent with the known ability of these reagents to disrupt myosin activity: RNAi constructs are the weakest, in part due to the long-half-life of targeted proteins (especially Zipper); spaghetti squash^AA^ blocks activation of myosin and has an intermediate effect, whereas Zipper^DN^ is a powerful dominant negative that prevents assembly of endogenous myosin II.

In order to reduce myosin II activity, we expressed UAS-spaghetti squash^AA^ or UAS-ROK RNAi in signal sending cells (SOP cells, using neuralized-GAL4) or in both signal sending and receiving cells (using pannier-GAL4) [29, 30]. Strikingly, reducing myosin II activity in signal sending cells was sufficient to decrease the rate of the Notch response in adjacent, wild-type neighbor cells (Fig. 1c, d; Additional file 1: Figure S1A-B), and in distant neighbors that signal to one another via basal actin-based protrusions (Fig. 1b, e, f; Additional file 1: Figure S1C). Similar effects were observed when myosin II activity was simultaneously compromised in both signal sending and receiving cells (Fig. 1g–i; Additional file 1: Figure S1D-E). In this case, the decrease in Notch response was more profound in distant neighbours. These data suggest that myosin II plays a critical role in Notch-Delta signalling, particularly between cells that contact one another via long, basal protrusions.

To test whether this represents a general role for myosin II in Notch signaling, we used a cell culture model of lateral inhibition [7]. This consists of a mixed populations of *Drosophila* S2R+ cells expressing either a synthetic Notch ligand or receptor. Once these form cell-cell contacts, myosin II is inhibited by pharmacological inhibitors or dsRNA-mediated knockdown of *zipper* or *spaghetti squash* expression (Fig. 1j–l) [31]. A luciferase-based transcriptional reporter is then used to measure Notch activity. Importantly, while acute treatment of the ROK inhibitor Y-27632 altered S2R+ cell shape, it did not change expression levels of ligand or receptor (Fig. 1j; Additional file 1: Figure S1F). Nevertheless, ligand-induced Notch signaling in this system was reduced by Y-27632 treatment in a dose-dependent manner (Fig. 1k; Additional file 1: Figure S1G). The role of cortical actomyosin-based tension in Notch signaling was confirmed using dsRNA-mediated knockdown of zipper or spaghetti squash expression (Fig. 1l). Moreover, by mixing control and dsRNA-treated cells, we showed that the maximal Notch response in this system requires myosin II in both signal sending and receiving cells. These findings support a general role for actomyosin contractility in driving a robust Notch response.

### Loss of myosin II activity disrupts bristle patterning

**Fig. 2:**
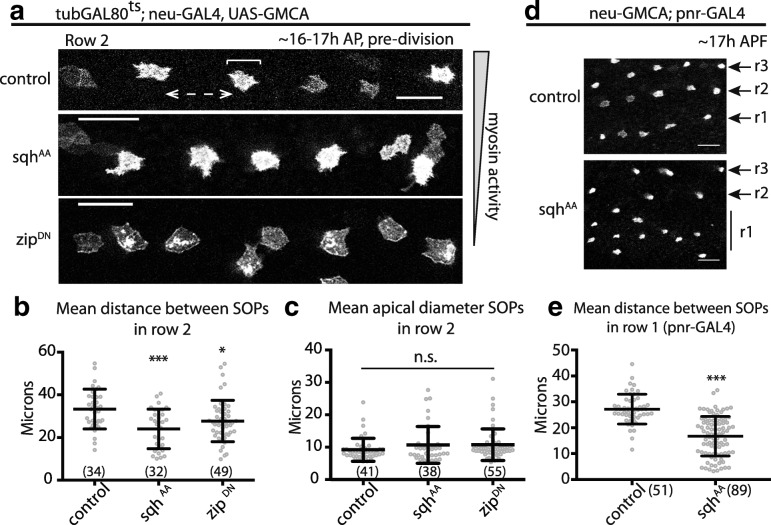
Changes in myosin II activity disrupt bristle patterning. Expression of phospho-insensitive MRLC (sqh^AA^) or dominant negative zipper (zip^DN^) constructs in (**a**) SOP cells only disrupt the final SOP pattern in the notum. Bristle row 2 is shown, control flies express UAS-LifeAct^Ruby^. (**b**) Mean ± SD distance between SOPs in bristle row 2 for the indicated genotypes (dashed, double-headed arrow in control (**a**)). **c** Mean ± SD apical diameter for SOPs in bristle row 2 for the indicated genotypes (see bracket in control (**a**)). **d** Expression of sqh^AA^ in all notum epithelial cells also disrupts the final SOP pattern in the notum. Bristle rows are indicated by R#, to the right of each panel. **e** Mean ± SD distance between SOPs in bristle row 1 for the genotypes in (**d**). Scale bars = 25 μm in all panels. ***p ≤ 0.001; *p ≤ 0.05; ns = not significant by one-way ANOVA with multiple comparisons. (*n*) = number of spaces (**b**) or cell diameters (**c**) measured. *N* ≥ 4 nota analyzed each genotype

We monitored bristle density in these flies to test whether these tension-dependent changes in the Notch response are sufficient to induce changes in lateral inhibition tissue patterning. Previous research had shown that decreased Notch signaling leads to both the formation of adjacent bristles and to an overall increase in the density of bristles [32]. The effect of non-muscle Myosin perturbations on patterning was much less severe than this. As a quantitative test of the impact of myosin on Notch-Delta-dependent lateral inhibition signaling, we measured the spacing between SOP cells in animals with decreased myosin II activity in signal sending cells (Fig. 2a). The expression of dominant negative myosin II, a treatment that leads to a strong reduction in myosin II activity, was associated with decreased spacing between SOP cells in rows, without altering SOP cell clustering (Fig. 2a–c). Similar results were seen in flies expressing *spaghetti squash*^*AA*^ in signal sending cells, leading to a reduction in pattern spacing (Fig. 2a–c). Furthermore, the expression of *spaghetti squash*^*AA*^ throughout the notum led to a decrease in bristle spacing (Fig. 2d, e)—a phenotype that was most evident in bristle row 1—but again, not to the formation of SOP cell clusters. Although we did not observe any disruption of the overall notum epithelium organization prior to cell division in animals expressing *spaghetti squash*^*AA*^ under pnr-GAL4, we cannot rule out subtle changes in this case or following the expression of *zipper*^*DN*^. Together, these data suggest actomyosin-based tension has a profound impact on long-range, protrusion-mediated Notch signaling without influencing signalling between cells that interact with one another via lateral contacts.

### The role of actomyosin contractility in the Notch signaling pathway

**Fig. 3:**
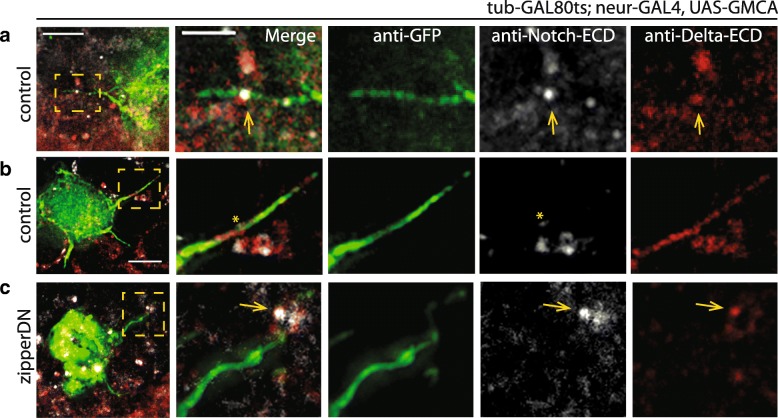
Localization of Notch and Delta. Fixed nota of the indicated GAL80, GAL4 genotypes crossed to **a**, **b** UAS-LifeAct^Ruby^ or **c** UAS-zipper^DN^ and co-immunostained for GFP (reporting filamentous actin), Notch extracellular domain, and Delta extracellular domain. Scale bar, 5 μm and 2 μm. See also Additional file 2: Figure S2

First, to ask how actomyosin contractility contributes to the activity of the Notch signaling pathway, we considered the possibility that non-muscle myosin II activity is required for the Notch receptor and/or Delta ligand to be presented on the surface of signal sending and receiving cells [17]. However, when we visualized Notch receptor and Delta ligands in SOP cells, we did not observe myosin-dependent changes in their localization (Notch - apical and basal puncta; Delta - cytoplasmic or basal puncta) or Delta (cytoplasmic or basal puncta) (Additional file 2: Figure S2). Notch and Delta were also observed along the length of protrusions regardless of myosin II status (*n* > 6 nota each genotype; Fig. 3a–c). Thus lowering myosin II activity does not appear to grossly affect the localization of Notch or Delta.

**Fig. 4:**
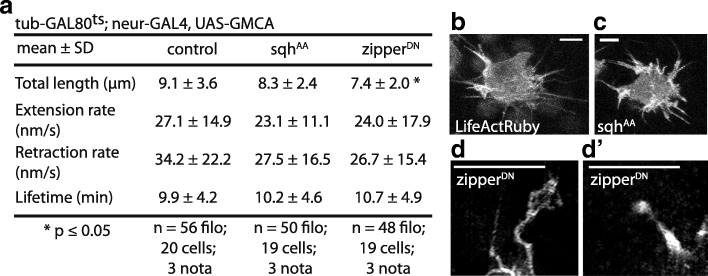
Effect of decreased myosin II activity on protrusion dynamics and morphology. (**a**) For the listed genotypes, we measured maximum protrusion length (from cell body to tip), rate of extension (time to maximum length), rate of retraction (time to disappearance into cell body), and protrusion lifetime. (**b**–**d**′) Live imaging of SOP cells in nota of the indicated genotype show that overexpressing sqh^AA^ does not appear to affect basal protrusion morphology, but overexpression of zipperDN does lead to abnormal protrusion shapes. Scale bars, 5 μm. *p ≤ 0.05, by unpaired *t* test. See also Additional file 3: Movie S1 and Additional file 4: Movie S2


Next, we asked whether myosin II activity has an impact on basal protrusion morphology or dynamics. This is important, since previous genetic and mathematical modeling data show that reductions in the length or dynamics (i.e., retraction, extension) of basal protrusions can lead to defects in long-range Notch signaling and, therefore, to decreased SOP cell spacing [33, 34]. We used time-lapse confocal microscopy to track the dynamics of basal protrusions in live SOP cells (Additional file 3: Movie S1) marked using cell-type-specific expression of the filamentous actin reporter GMCA, the GFP-tagged actin binding domain of Moesin (Fig. 4) [35]. Basal protrusions exhibited cycles of extension and retraction with a period of ~ 10 min, with an average maximum length of 10 μm, consistent with previously published data [33]. Protrusions changed shape prior to and during retraction (Additional file 3: Movie S1) through a process reminiscent of helical buckling observed in mechanically active filopodia in cell culture [36]. Importantly, however, the behavior of protrusions appeared similar in both control and *spaghetti squash*^*AA*^ expressing tissues (Additional file 4: Movie S2), and their morphology was unaffected by the expression of *spaghetti squash*^*AA*^ (Fig. 4a–c). However, when we induced a strong loss of myosin II activity via expression of the dominant negative construct, we observed changes in the length and morphology of protrusions (e.g., bulbous tips, fanning) (Fig. 4d, d’), similar to those seen following myosin inhibition in cell culture [37]. We conclude that while decreased myosin II activity can lead to protrusion defects, the incidence of these defects does not correlate with the reduction in protrusion-mediated long-range Notch signaling that we observed in animals expressing *spaghetti squash*^*AA*^.

Additional file 3: **Movie S1.** Basal protrusion dynamics in a control SOP cell. SOP cell expressing UAS-GMCA under the neur-GAL4 driver. Movie represents a maximum projection over 2.1 μm. Time in seconds. Scale bar, 10 μm. (AVI 4726 kb)



Additional file 4: **Movie S2.** Basal protrusion dynamics in a SOP cell with decreased myosin activity. SOP cell expressing UAS-GMCA and UAS-spaghetti squash^AA^ under the neur-GAL4 driver. Movie represents a maximum projection over 2.1 μm. Time in seconds. Scale bar, 10 μm. (AVI 8361 kb)


### Actomyosin contractility and endocytosis influence Notch signaling in distinct ways

How might the actomyosin cortex influence Notch signaling? Endocytosis requires deformation of the cell cortex, whose stiffness is regulated by actomyosin contractility [38, 39]. At the same time, the forces generated during ligand internalization have been proposed to drive the mechanical activation of Notch receptor [7, 9, 40]. This made it important to test whether the impact of actomyosin contractility on signaling and on lateral-inhibition patterning in vivo functions through its effects on endocytosis.

To do so, we first examined the ability of cells with reduced myosin II activity to endocytose Delta ligand using a ligand uptake assay [41]. Delta-positive puncta were observed in both control and *zipper*^*DN*^ expressing cells without these being a visible difference in the numbers of these puncta (Additional file 5: Figure S3A).

**Fig. 5:**
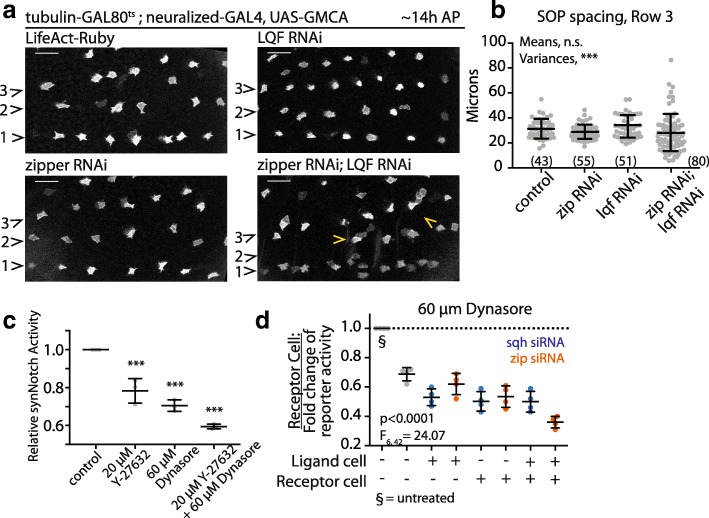
Overlapping roles of endocytosis and myosin II activity for Notch activation. **a** SOP cell patterns for pupae of the indicated genotypes. Bristle rows 1–3 shown (indicated to the left of each panel). Scale bars, 25 μm. **b** Mean ± SD distance between SOP cells along bristle row 3 at 14 h AP for genotypes in (**a**); (*n*) = pairs measured, *N* ≥ 3 pupae per genotype. n.s. by one-way ANOVA. ***p < 0.0001 by Brown-Forsythe test, F_3, 225_ = 9.71. (**c**) synNotch activation in S2R+ heterogeneous populations treated with y-27632 alone, dynasore alone, or y-27632 and dynasore together. Mean ± SEM shown, ***p ≤ 0.001 by ANOVA with multiple comparisons. **d** Relative levels of synNotch activation in response to siRNA against zipper (orange) or spaghetti squash (blue) in the presence of 60 μM dynasore. Plus sign indicates transfection with siRNA, Minus sign indicates transfection with control siRNA targeting the white gene. Mean ± SEM for 4 experimental repeats shown. *p* values determined by two-way ANOVA. Interaction term is not significant (*F*_6,42_ = 1.62). See also Additional file 5: Figure S3

We next used dsRNA to silence known regulators of Delta endocytosis and myosin II activity to determine how the two effects combine to influence long-range Notch signaling. Decreased Delta endocytosis in SOP cells, induced by RNAi-mediated silencing of the epsin liquid facets (lqf) [42], led to a slight increase in the numbers of SOP cell clusters (10.7 ± 3.8 SOP pairs per LqfRNAi nota vs 4.5 ± 1.8 SOP pairs per control nota, *N* ≥ 3 nota each genotype; Additional file 5: Figure S3B-E), which is consistent with the requirement for epsins in Notch-Delta signalling (Note this phenotype differs from that seen following reductions in myosin II activity) [40, 42]. We then combined these perturbations While the animals expressing dsRNAs that target each system alone had weak bristle patterning defects (Fig. 5a), when we co-expressed *zipper-* and *lqf-RNAi* in SOP cells (using tubulin-GAL80^ts^ to temporally control RNAi expression so that the animals remained viable), we observed severe patterning defects. These appeared to be a combination of the two phenotypes observed in the single mutants, i.e., there was an increase in the variability of bristle spacing together with an enhanced number of GFP-positive cell clusters relative to the expression of either RNAi alone (Fig. 5a, b; Additional file 5: Figure S3F). These data support the idea that Delta ligand endocytosis and myosin II activity act in distinct ways to impact Notch signaling in vivo via lateral and protrusion-mediated signaling, respectively.

This result was further supported by experiments performed in cell culture using the synthetic Notch system, where we perturbed both myosin II activity and endocytosis via dsRNA and pharmacological inhibition (Fig. 5c-d; Additional file 1: Figure S1H). As previously published, Dynasore, an inhibitor of dynamin-mediated endocytosis [43], decreases Notch activation in this system [7]. Strikingly, when Dynasore was used in combination with Y-27632, we observed a further reduction in Notch response (Fig. 5c). Moreover, additive effects were observed in experiments in which we used dsRNAs to target either *zipper* or *spaghetti squash* expression in signal sending or receiving cells in the presence or absence of Dynasore (Fig. 5d). Again, these data point to myosin II activity and endocytosis having distinct functions in the regulation of Notch signaling.

### Interacting protrusions are under mechanical tension

**Fig. 6:**
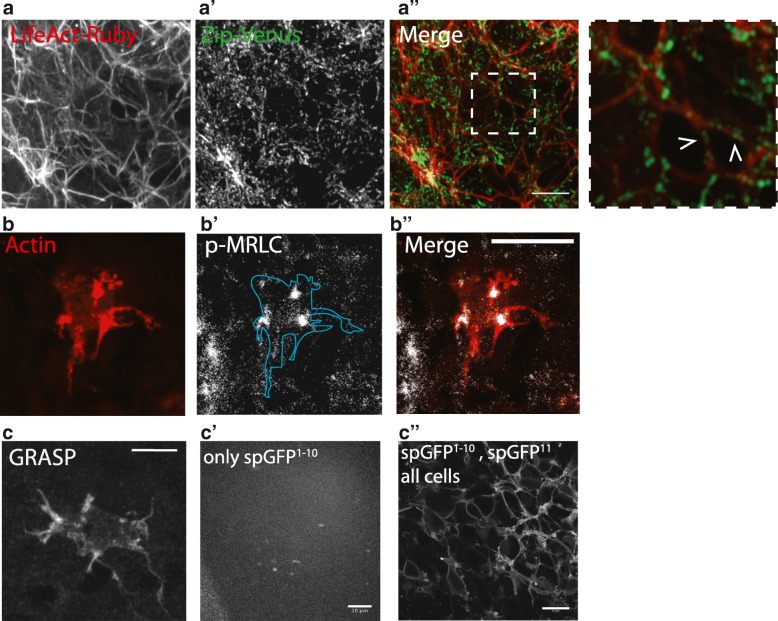
Protrusion contacts and Myosin II localization. (**a**–**a**″) Filamentous actin in the basal protrusions co-localizes with myosin II heavy chain (visualized with Venus) in notum explant. Images are a z-projection ~ 2 μm basal to the cytoplasm of notum epithelium, where basal protrusions dominate. (**b**–**b**″) Phosphorylated MRLC (p-MRLC, spaghetti squash) localizes to the cell-proximal base of basal protrusions in fixed nota. (**c**–**c**″) Image from a fixed nota of the indicated genotype, GRASP (reconstituted membrane bound GFP) indicates the extent of cell-cell contacts between signal sending (pictured) and receiving cells. (**c**) A single SOP cell expressing GFP^11^ with neighboring epithelial cells expressing GFP^1–10^. (**c′**) A single SOP cell expressing GFP^11^ with neighboring epithelial cells *not* expressing any GFP^1–10^. (**c″**) Expression of both GFP fragments in all notum cell types. Scale bars: for **b**″, **c** = 5 μm; for **a**″, **c**′**c**″ =10 μm. See also Additional file 6: Figure S4

While the apical actomyosin cytoskeleton is known to control many aspects of notum cell biology [21, 30, 44], little is known about its impact on basal protrusions. If myosin II were to contribute to Notch signaling by enabling protrusions that make adhesive contacts to exert forces on one another, several preconditions would have to be met. Myosin II would have to be (i) present and active within the basal domain of cells and (ii) generate pulling forces between these protrusions. When we imaged myosin II in the basal-most focal plane of the tissue using the Venus-tagged myosin II heavy chain gene trap [45], myosin II was visible along the length of protrusions and at their base, where it co-localized with filamentous actin (Fig. 6a–a″). In order to determine whether this basal myosin II pool is likely to generate forces we imaged phosphorylated endogenous RLC (pMRLC, phospho-myosin regulatory light chain [27]). We observed pMRLC (and thus active myosin II) localized in clumps at the base of protrusions in SOP cells (Fig. 6b–b″; Additional file 6: Figure S4A). These data are consistent with models in which the contractile actomyosin meshwork at the base of cellular protrusions contributes to retrograde flow, amplifying actin treadmilling within the protrusion [46, 47].

Having established the presence of myosin II in and at the base of protrusions, we wanted to assess the extent of contacts between the basal domain of cells in the notum. These can be visualized using the GRASP system (GFP reconstitution across synaptic partners) [48]. Membrane-bound GFP^1–10^ was expressed in signal receiving cells (via pnr-GAL4) and GFP^11^ in signal sending cells (via ase-LexA, with LexAOp-GAL80 to inhibit GAL4 activity), so that the reconstituted GFP fluorescence would be visible only when the two cell types came into contact with one another (Fig. 6c; Additional file 6: Figure S4B). In control tissues expressing GFP^1–10^, we observed background levels of fluorescence (Fig.6 c′). In control tissues where all cells express both GFP^1–10^ and GFP^11^, we observe that all cell membranes fluoresce, including the nuclear envelope (Fig. 6c″). However, when we assessed the ability of single GFP11 cells to contact their neighbours, we saw that basal cell-cell contacts are not restricted to protrusion tips, but are extensive and run along the length of the protrusions (Fig. 6c). These data indicate that protrusions form a network of basal cell-cell contacts that effectively extend the range of physical contact between cells in the notum to 2–3 cell diameters, as suggested previously [33].

**Fig. 7:**
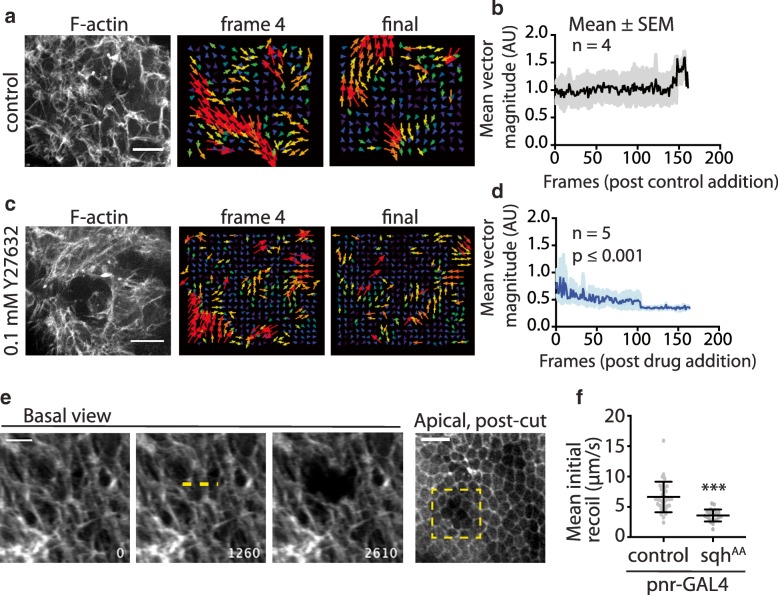
Basal protrusions and mechanical force. PIV analysis was performed after addition of either (**a**, **b**) control (dH_2_O) or (**c**, **d**) 0.1 mM Y27632. (**b**, **d**) Quantification of mean vector magnitude for each frame pair (1 frame captured every 10 s) after control or drug addition. Comparison of best-fit slope relative to control, ***p < 0.0001. **e** Basal view of live nota expressing UAS-LifeActRuby under pnr-GAL4 in all signaling cells during a laser ablation protocol. Dashed yellow line indicates location and length of cut target. Time, in ms, at lower right. Apical view post-cut indicates that the apical regions of cells were not affected in the area in which the basal surface was targeted (yellow box). Scale bars: basal view, 10 μm; apical view, 25 μm. **f** Quantification of mean initial recoil post-laser ablation of the basal protrusion network for control (UAS-LifeActRuby; *n* = 35 cuts; 10 pupae) or spaghetti squash^AA^ (*n* = 22 cuts; 8 pupae) expressing nota. ***p ≤ 0.001 by unpaired *t* test. See also Additional file 7: Movie S3


In line with this, when we imaged the full set of protrusions in vivo using *pannier-GAL4* to express UAS-LifeAct^Ruby^ in both signal sending and receiving cells in the notum, we observed a complex network of protrusions that criss-crossed the basal surface of the epithelium (Fig. 7a). These networks fluctuate in density over time (Additional file 7: Movie S3). During the window of observation, particle image velocimetry (PIV) [49] revealed that the basal domain is in constant flux (Fig. 7a, b), without there being evidence for tissue-wide polarity. Importantly, this movement was dependent on actomyosin contractility, since it was markedly reduced by treatment with Y-27632 [29]. Acute treatment with Y-27632 led to a steady decrease in the pulsatile movement of basal cell-cell contacts over a period of a few minutes (Fig. 7c, d). These data suggest that basal contacts transmit forces between cells which depend on myosin II.

Additional file 7: **Movie S3.** Basal protrusion dynamics in notum explant. Nota expressing UAS-LifeAct-Ruby under the pnr-GAL4 driver to visualize filamentous actin in all epithelial cells. This movie is from a tissue explant cultured in Clone8 medium. Movie represents a single z-plane, time stamp: (minutes to seconds). Scale bar, 10 μm. (AVI 1802 kb)


Finally, to test whether these basal protrusion contacts are subject to mechanical tension tension, we performed laser ablation to disrupt them. Using a focused UV-laser, we made ~ 10-μm cuts within the basal surface of the notum, disrupting the protrusion network without affecting more apical planes (Fig. 7e). Cuts induced a rapid recoil, followed by a slower relaxation, as expected for tissues under tension. When we expressed the *spaghetti squash*^*AA*^ construct under the pnr-GAL4 driver to reduce myosin II activity throughout the notum, we observed a twofold decrease in the mean initial recoil velocity, from 6.6 ± 2.5 μm/s (mean ± SD, *n* = 35 cuts, 10 pupae; Fig. 7f) to 3.6 ± 1.0 μm/s (*n* = 22 cuts, 8 pupae; *p* < 0.0001, unpaired *t* test). Taken together, these data support a model in which myosin II-dependent tension in basal contacts that connect epithelial cells in the notum facilitate robust activation of Notch response during pattern formation in the notum.

## Discussion

In *Drosophila*, the pattern of bristles on the dorsal thorax of the fly is set up by Notch-Delta signaling through a process of lateral inhibition [50]. As we show here, this signalling is facilitated by both actomyosin contractility and Epsin-mediated endocytosis [14]. While a reduction in myosin II activity in signal sending cells is not sufficient to induce adjacent cells to adopt the SOP cell fate, in combination with reductions in Epsin function, we observe frequent SOP cell clusters. This suggests that Notch signaling in adjacent neighbors depends on both myosin II-mediated contraction and Epsin-mediated endocytosis forces. The two systems could well work in tandem, since cortical actomyosin may function as a platform that the endocytic machinery can use to exert forces. However, over longer distances, where lateral inhibition signaling relies on protrusions, myosin II acts alone. Here, reductions in myosin II activity are sufficient to lead to aberrant cell fate decision making and decreased spacing between SOP cells. For this model of Notch activation to work [33, 34, 51, 52] would require that (i) Notch and Delta localize to protrusions; (ii) that protrusions have physical interactions; (iii) that these protrusion interactions are subject to tension, in order to activate Notch receptor. Here we show that each of these conditions is met.

How might Notch receptor be activated on protrusions? Our data suggests that one way this could occur is through the contact, engagement, and retraction of basal protrusions in Notch and Delta expressing cells. Importantly, since the contact area between protrusions can be extensive, Notch/Delta signaling molecules localized along the protrusions could potentially contribute to signal activation. In addition, when contact has been made, the ligand and receptor could diffuse along the length of the protrusion and become trapped at the site of contact—as suggested previously [53]. Once Notch and Delta have become engaged, actomyosin-dependent extension/retraction cycles could provide shear forces parallel to the plasma membrane that contribute to the activation of Notch. Alternatively, Notch could be activated by the simple pulling of protrusions on one another. Filopodia are able to exert pulling forces on their environment up to ~ 1 nN [36, 54], which is more than sufficient to activate Notch receptor and much higher than the forces associated with endocytosis [40, 55]. One caveat of our work is that, although we did not observe any change in basal Notch or Delta localization in cells with lowered levels of actomyosin contractility, due to the relatively low levels of basally localized Notch and Delta, we cannot rule out an effect. Development of next generation Notch activity reporters that allow the rapid visualization of local receptor activation in vivo will be necessary to reveal the mechanical details of Notch signaling in cellular protrusions.

While our data do not rule out the possibility that endocytosis contributes directly to protrusion-mediated Notch activation. However, the structure of filopodial-like protrusions, with bundled actin closely associated with the plasma membrane, may be refractory to endocytosis. Indeed there is evidence for the existence of endosomal ‘hot spots’ at the base of cellular protrusions rather than along them [56]. A closer analysis of the ultrastructure of basal protrusions could help to reveal the impact of local structure on the numbers of endocytic pits formed.

How much does protrusion-mediated Notch-Delta signaling contribute to the cell fate decision making process of epithelial cells? Previously, it was shown that stochastic noise can be a feature of signaling mechanisms and contribute to the plasticity of bristle patterning [57]. More generally, stochastic amplification of noisy signals, especially as part of signaling feedback loops, can drive bistable systems in both models and biological systems [58–61]. This means that the amount of signaling protein present on basal protrusions is likely sufficient to induce changes in cell fate: when two cells beginning to downregulate Notch response and upregulate pro-neural genes are “too close,” i.e., within range of each other’s basal protrusions, we commonly observe switching away from the SOP fate in at least one of them. Thus, the long-range Delta signal, amplified through the feedback mechanisms which exist to reinforce Notch-mediated cell fate decisions [62, 63], is likely sufficient to impose the epithelial fate, even if these proteins are found concentrated at lateral cell-cell contacts [33, 64].

Recent evidence shows that the patterning of bristle rows begins developing in the notum far earlier than 12 h APF, where we begin our analysis [65]. Although we have observed basal protrusions in fixed nota as early as 9 h APF (unpublished data), technical challenges prevent us from being able to perform our live analysis of Notch dynamics prior to 12 h APF. Corson et al. also suggests that bristle patterning terminates prior to 12 h APF; however, our data clearly show that completion of lateral inhibition-dependent decision making is, at least in some cells, a temporally extended process [66] and these fate choices remain plastic as late as 12 h APF in wild-type animals. Given that myosin II is able to alter Notch-Delta signaling in cell culture and in vivo, and given the prevalence of protrusions in biological systems [67], it will be important in the future to determine whether actomyosin-dependent protrusion-mediated Notch signaling plays much more general roles in tissue development and homeostasis than currently appreciated.

## Conclusion

Our results show that actomyosin contractility plays a role in Notch signaling both in a cell culture model of signaling and in bristle patterning in the developing fly. By tuning the activity of myosin II to affect contractility without affecting protrusion dynamics and morphology, we show that protrusion interactions are subject to contractile forces and that myosin II activity is required for robust signaling between cells whose only contact is via basal protrusions. Our data also show that, actomyosin contractility in cells that contact one another through extensive lateral contacts promotes the Notch. In these cells endocytosis and myosin-dependent pulling may both contribute to force-dependent Notch activation. These results add to our understanding of protrusion-mediated signaling in the patterning of self-organized tissues. Importantly, they also suggest a new mechanism of mechanotransduction via which forces generated within the actomyosin cytoskeleton impact signaling to help pattern a tissue.

## Methods

### Fly strains

*GAL4 Drivers*: tub-GAL80^ts^; neu-GAL4, UGM. Neu-GMCA; pnr-GAL4. N^sfGFP^; neu-GAL4. N^sfGFP^; pnr-GAL4. shotgun^GFP^; pnr-GAL4. shotgun^GFP^, neu-GMCA; neu-GAL4. UAS-lifeAct^Ruby^; pnr-GAL4. *UAS Responders:* UAS-zipper^dn, gfp^. UAS-squash^AA^. UAS-LifeAct^Ruby^. UAS-liquid facets RNAi. UAS-ROK RNAi. UAS-white RNAi UAS-zipper RNAi. *Other*: zipper^Venus, Flag^. *GRASP*: ase-LexA-p65/CyO(Kr:GFP); LexAOp-GAL80, UAS-CD4spGFP(1–10)/TM6BTb. LexAOp-CD4spGFP(11)/CyO; pnr-GAL4/TM6BTb.

### Microscopy

White pre-pupae were picked and aged to ~ 12–24 h AP at 18 °C. Live pupae were removed from pupal case and mounted on a slide as previously described [68]. Final patterns, live imaging of N^sfGFP^, ex vivo experiments, and filopodia imaging were performed on either a Leica SPE confocal, × 40 oil immersion objective (1.15 NA) at room temperature or a Nikon EclipseTi, × 20 (0.75 NA) or × 60 (1.4 NA). Localization of myosin in live pupae was performed on a Zeiss LSM880 with AiryScan, × 63 (1.4 NA) oil immersion objective. Fixed nota were imaged on a Leica SPE confocal, × 63 (1.3 NA) oil immersion objective. Fixed SIM images were obtained on a Zeiss Elyra PS.1, × 63 (1.4NA) oil immersion.

### Laser ablation

Live images were acquired on a Zeiss Axio Imager.M2 m, × 40 (1.2NA) water objective, using Micromanager (Vale Lab) acquisition software. An Nd:YAG UV laser (Continuum) was interfaced with the confocal microscope to allow steered laser incisions [69]. We made ~ 10-μm incisions orthogonal to the anterior-posterior axis at a laser power ~ 3.0 μJ.

### Immunofluorescence

*Primaries*: chicken anti-GFP (1:1000, Abcam ab13970, RRID:AB_300798); rabbit anti-pS19-MRLC (1:50, CST 3671); mouse anti-Delta extracellular domain (1:100, DSHB c594.9b, RRID:AB_528194); mouse anti-Notch extracellular domain (1:200, DSHB c458.2H, RRID:AB_528408); guinea pig anti-Delta ECD (1:2000, M. Muskavitch). *Secondaries*: Rhodamine Red-X anti-guinea pig (1:2000, Jackson ImmunoResearch Labs #106-295-003, RRID:AB_2337428) and Cy5 anti-mouse (1:2000, Jackson ImmunoResearch Labs #115-175-146, RRID:AB_2338713); AlexaFluor 488 anti-chicken (1:1000, ThermoFisher Scientific A-11039, RRID:AB_2534096) and AlexaFluor 568 anti-rabbit (1:1000, ThermoFisher Scientific A-11011, RRID:AB_143157); Texas Red-X Phalloidin (1:500, ThermoFisher Scientific T-7471) were used to visualize F-actin. Nota were mounted in 50% glycerol or Vectashield (with DAPI, Vector Laboratories) for labeling nuclei.

### Ex vivo

Live nota were dissected and attached to 35-mm^2^ glass bottom dishes (Matek) using a thrombin/fibrinogen (Sigma) clot, then cultured in 250-μL modified Clone8 medium (Schneider’s insect medium (Sigma), 2.5% fly extract, 2% fetal bovine serum (ThermoFisher Scientific) as previously described [68]. After initiation of imaging, either control (250 μL Clone8 + 0.5 μL dH_2_O) or 0.1 mM Y27632 (250 μL Clone8 + 0.5μL 100 mM Y27632; Sigma) was added to the dish.

### Molecular cloning

Long hair-pin dsRNA was designed using SnapDragon (DRSC/TRiP Functional Genomics Resources, https://www.flyrnai.org/cgi-bin/RNAi_find_primers.pl). Gene-specific amplicons of *sqh*, *zipper*, and *white* genes were amplified from fly genome by PCR, inserted into pDONR221 vector, and subcloned into pWALIUM10 RNAi vector. All constructs verified by sequencing. The primers used for the synthesis of dsRNA were as follows (F, forward; R, reverse), sequence 5′-3′:

- *sqh* F: GGGGACAAGTTTGTACAAAAAAGCAGGCTTCCGGCTCCATTTAGCTCCATTA
- *sqh* R: GGGGACCACTTTGTACAAGAAAGCTGGGTGGCAGGACGCCCATATTCTC
- *zippe*r F: GGGGACAAGTTTGTACAAAAAAGCAGGCTTCCACAGGGTACAGCCGATAAA;
- *zipper* R: GGGGACCACTTTGTACAAGAAAGCTGGGTGCTTGTGCTTGCAGCTTCTTC;
- *w* F: GGGGACAAGTTTGTACAAAAAAGCAGGCTTCTGCCCAGTGTCCTACCA;
- *w* R: GGGGACCACTTTGTACAAGAAAGCTGGGTGTACGAGGAGTGGTTCCTTGA.

Primers used for qPCR:

- GAPDH F: CCAATGTCTCCGTTGTGGA
- GAPDH R: TCGGTGTAGCCCAGGATT
- sqh F1: CGAGGAGAATATGGGCGTCC
- sqh R1: CCTCCCGATACATCTCGTCCA
- zipper F: CCAAGACGGTCAAAAACGAT
- zipper R: GATGTTGGCTCCCGAGATAA

### Cell culture

Drosophila S2R+ cells were grown in Schneider’s Drosophila media (Gibco) supplemented with 10% fetal bovine serum and 0.5% Pen/Strep (Gibco). Cells were transfected using Effectene transfection reagent (Qiagen) [70], with DNA mixture containing: 0.1 μg pAct-Gal4, 0.4 μg pUAST-dsRNA, 0.01 μg pUbi::GBN-Notch-QF, 0.09 μg pQUAST-luciferase (for signal receiving cells); 0.1 μg pAct-Gal4, 0.4 μg pUAST-dsRNA, 0.1 μg pUbi::GFP-mcd8-Ser (for signal sending cells). Transfected cells were cultured for 10 days to allow efficient knock-down of target genes. Signal sending and receiving cells were washed twice with fresh culture medium, suspended, and mixed together in 1:1 ratio. Cells were cultured for an additional day before screening luciferase activity (Steady-Glo Luciferase Assay Kit, Promega) using a SpectraMax Paradigm Multi-Mode Microplate Reader. For Dynasore treatment, transfected cells were washed twice by culture medium, incubated with medium containing 60 μM Dynasore and/or 20 μM Y-27632 (final concentration) for 1 h. Cells were resuspended, mixed in 1:1 ratio, and cultured for an additional day in the presence of Dynasore before luciferase assay. Knock-down efficiency was tested by qPCR. Real-time PCR was performed using iTaq SYBR Green Supermix (Bio-Rad) with GAPDH as a control.

*qPCR:* S2R+ cells plated in six-well plate were transfected with indicated dsRNAi plasmid (0.6 μg) together with pAct-GAl4 (0.2 μg) using Effectene. dsRNA against *white* used as control. The transfection procedure was repeated two more times every 4 days to maximize transfection efficiency. Total RNA was extracted from S2R+ cells after the 3rd transfection using TRIzol Reagent (ThermoFisher Scientific). Raw RNA was treated with DNase I, purified by QIAGEN RNeasy kit, and converted to cDNA template using iScript cDNA Synthesis Kit (Bio-Rad). *Y27632 treatment*: Cells expressing either synNotch or GFP-ligand were treated with 20 μM Y-27632 for 1 h and then mixed and cultured in the presence of 20 μM Y-27632 for 1 day before analysis.

### Quantitative analysis and statistics

All statistical analysis was performed in Prism 6 (GraphPad). Specific tests used are indicated in the text and figure legends, see Additional file 8 for raw data.

Figure 1: N^sfGFP^ signal was measured as previously described [24]. Briefly, the N^sfGFP^ transgene includes a nuclear localization signal. GFP fluorescence localized to nuclei either adjacent to (1 N) or one cell removed (2 N) from an SOP (which lack GFP expression) was measured by drawing an ROI in the nucleus and recording mean intensity for the ROI for each time point until nuclear envelope breakdown (NEB). Since epithelial cells are staggered in their cell fate decision making process, and we previously established that exit from G2 phase of the cell cycle is Notch signaling dependent, we use NEB as a fixed time point for comparison. For the purposes of these graphs, *t* = 0 at NEB. The data was normalized to the fluorescence at the first time point of the movie (i.e., time point closest to 12 h APF).

Figures 2 and 5: Mean distance and apical diameter between SOPs was measured in bristle row 2: an unprojected, z-slice image is rotated to align bristle row 2 with 0° axis, an ROI is drawn to encompass all SOPs in row 2, and the ROI is collapsed along the *y*-axis to result in a 1D histogram along the bristle row (*x*-axis). Distance between SOPs is defined as the centroid to centroid distance between signal peaks along the histogram; apical diameter is defined as the edge-to-edge measurement of a signal peak (i.e., where signal reaches 0). To verify that we were only measuring the distance between true SOP cells, we captured 12 h time-lapse movies (starting at 12 h APF). We therefore measured the distance between GFP+ cells which become SOPs and not GFP+ cells that switch back to epithelial fates. However, in measuring grouping, we measured any GFP+ cells adjacent to each other.

Figure 4: Live images were maximum projected. Total length = maximum length from cell-proximal base to tip; extension rate = time for a protrusion to appear until it reaches maximum length; retraction rate = time for a protrusion to disappear after reaching maximum length; lifetime = total time during which a protrusion is visible.

Figure 7: Particle image velocimetry (PIV) was performed using the FIJI plugin described in [49]. Mean vector magnitude (second iteration) for the field of view for each time point pair was calculated. The region shown in the micrograph is the full extent of the analyzed area. We chose ROI of consistent area to measure movement. We picked areas of the excised nota that were relatively flat and close to the coverslip—due to the nature of the experiment excised nota sometimes curl up or fold, and we wanted to measure movement in contiguous regions of tissue. Mean initial recoil is measured by the average movement/time of ≥ 2 fiducials before and after laser ablation and was performed using the manual spot tracker in Icy [71].

Supplement: Filopodia dynamics were measured by manually tracing protrusions in z-projected time-lapse images. Myosin localization: basal images (up to 1 μm maximum projection). ROIs were drawn to include only basal filopodia areas. All other measurements were performed as described in main text figures.

## Additional files

Additional file 1: **Figure S1.** Contribution of Myosin II activity to Notch response. (A) RNAi against an activator of Myosin II activity, Rho kinase (ROK) in signal sending cells alone does not disrupt protrusion morphology. (B-C) Decreased ROK activity in signal sending cells alone (via neur-GAL4) leads to decreased Notch response in both (B) adjacent and (C) distant wildtype neighboring cells (non-linear regression, comparison of fit, Prism). (D) Decreased ROK activity in all cells (via pnr-GAL4) does not affect the rate of signaling between adjacent cells, but does decrease the total signal (1 N control vs RNAi elevations, *p* < 0.001 by linear regression). (E) The rate of Notch response in distant neighbors is significantly affected by ROK RNAi expression (linear regression, Prism). (F) S2R+ cells expressing synNotch in the absence of ligand expressing cells and cultured in the presence of Y27632 and/or Dynasore do not exhibit changes in their expression of synNotch in response to drug treatment. (G) Acute inhibition of ROK does not significantly alter the basal synNotch activity measured in S2R+ cells expressing synNotch in the absence of ligand expressing cells. (H) Basal synNotch activity is affected by transfection of zip and spaghetti squash siRNA, but not by acute treatment with Dynasore. However, because fold changes of synNotch activity in the presence of GFP-ligand is calculated based on each treatment respectively, the relative fold changes should still primarily reflect the efficiency of synNotch cleavage under different conditions. (PDF 1657 kb)

Additional file 2: **Figure S2.** Localization of Notch and Delta with decreased Myosin II activity. In tissues expressing control (LifeActRuby) or sqh^AA^ constructs in SOP cells (tubGAL80^ts^; neur-GAL4, UAS-GMCA) we observe no differences in (A) Notch localization or (B) Delta localization at a single apical section and basal projection (over 2 μm). Scale bars, 10 μm and 5 μm (sqh^AA^ anti-Delta panels). (PDF 4856 kb)

Additional file 5: **Figure S3.** Role of ligand endocytosis in Notch signaling. (A) Delta ligand endocytosis assay in SOP cells expressing LifeAct^Ruby^ (control) or zipper^DN^ under tubGAL80^ts^; neur-GAL4, UAS-GMCA. Endocytosed Delta is specifically visualized through uptake of the anti-Delta (ECD) monoclonal antibody, red puncta (arrows). GFP is stained to visualize the SOP cell body. We find no significant difference in numbers of Delta positive puncta between the control or myosin II perturbed genotypes (graph). (B-C) RNAi against key regulator of ligand endocytosis pathway (liquid facets) lead to a pairing phenotype (indicated by yellow brackets). Pairs of neuralized expressing cells are seen with less frequency in control pupae. (B-C) Scale bars, 25 μm. (D-E) Quantifications of patterns for genotypes in (B-C). (D) Apical ‘cell’ diameter, measuring grouping/pairing. ***p < 0.001 by unpaired t-test. (E) Measurements of the distance between neuralized expressing cells. ***p < 0.001 by unpaired t-test. (n), number of distances measured, *N* ≥ 3 nota measured for each genotype at ~ 14 h AP. (F) Grouping is observed in cells co-expressing RNAi targeting zipper and liquid facets in SOP cells, compared to either RNAi expressed in SOP cells alone. We note that the grouping phenotype is slightly different in neur-GAL4, UAS-GMCA (D) and tubGAL80^ts^; neur-GAL4, UAS-GMCA (F) background, for unclear reasons. (PDF 5104 kb)

Additional file 6: **Figure S4.** Data supporting main Fig. 6. (A) additional panels of the same genotype and treatment as in Fig. 6B-B”. Filamentous actin in red, phosphorylated myosin regulatory light chain in greyscale. Basal projection images are a maximum intensity projection over 2–4 μm to visualize protrusions. Single slice images are a single z-plane with in the projection images that show the pMRLC puncta. (B) Cartoon to clarify the genetics of the GRASP experiment in Fig. 6C-C”. (PDF 3497 kb)

Additional file 8: Raw data file. Data analyzed for the main and supplemental figures. (XLSX 245 kb)
