# Supplementary figures and images for: A role for actomyosin contractility in Notch signaling
Source: BMC Biol. 2019 Feb 11;17:12. doi: 10.1186/s12915-019-0625-9 (PMC6369551; doi:10.1186/s12915-019-0625-9)

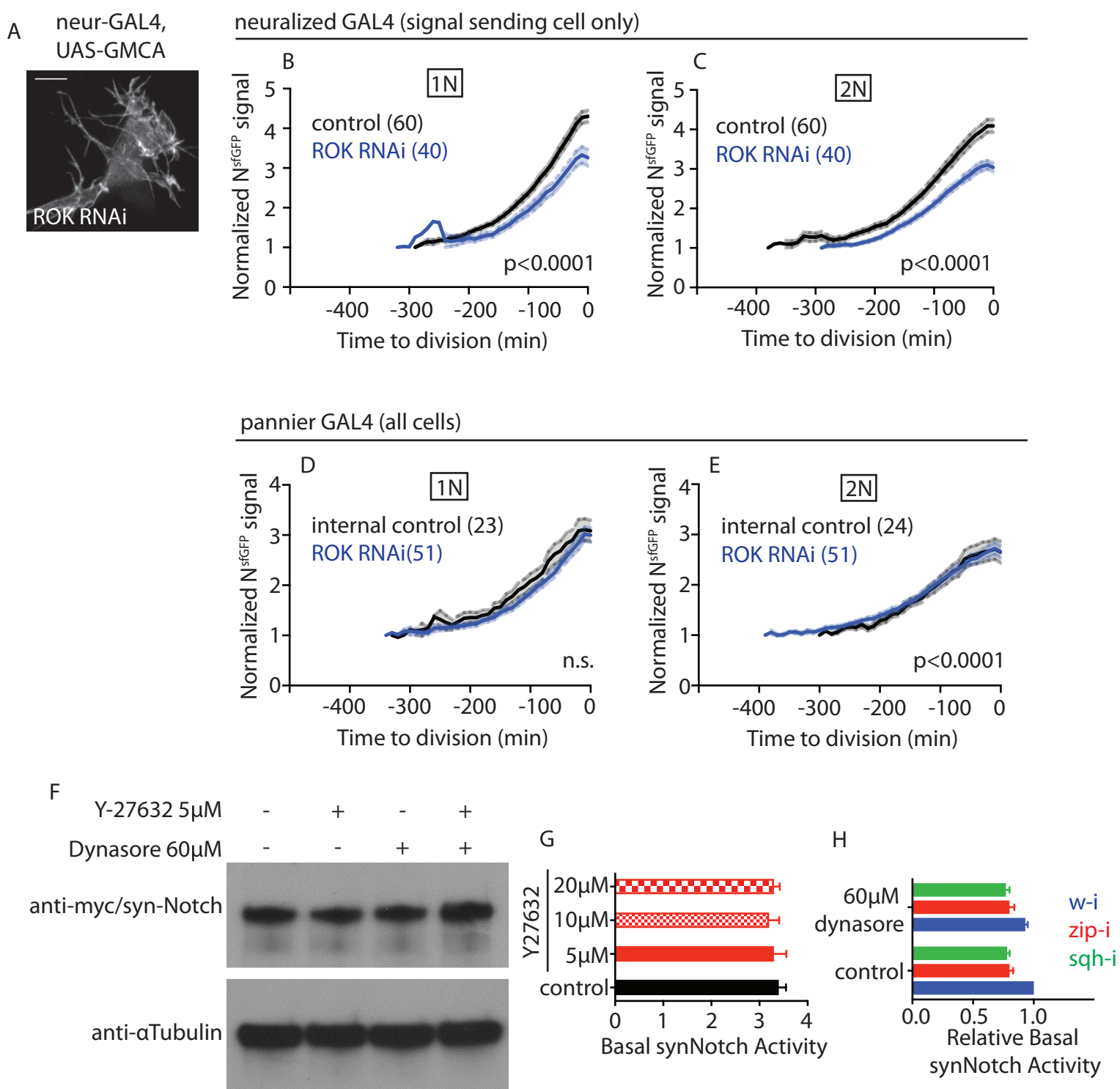

Supplement: Supplementary file 4 — Figure S1. Contribution of Myosin II activity to Notch response. (A) RNAi against an activator of Myosin II activity, Rho kinase (ROK) in signal sending cells alone does not disrupt protrusion morphology. (B-C) Decreased ROK activity in signal sending cells alone (via neur-GAL4) leads to decreased Notch response in both (B) adjacent and (C) distant wildtype neighboring cells (non-linear regression, comparison of fit, Prism). (D) Decreased ROK activity in all cells (via pnr-GAL4) does not affect the rate of signaling between adjacent cells, but does decrease the total signal (1 N control vs RNAi elevations, p < 0.001 by linear regression). (E) The rate of Notch response in distant neighbors is significantly affected by ROK RNAi expression (linear regression, Prism). (F) S2R+ cells expressing synNotch in the absence of ligand expressing cells and cultured in the presence of Y27632 and/or Dynasore do not exhibit changes in their expression of synNotch in response to drug treatment. (G) Acute inhibition of ROK does not significantly alter the basal synNotch activity measured in S2R+ cells expressing synNotch in the absence of ligand expressing cells. (H) Basal synNotch activity is affected by transfection of zip and spaghetti squash siRNA, but not by acute treatment with Dynasore. However, because fold changes of synNotch activity in the presence of GFP-ligand is calculated based on each treatment respectively, the relative fold changes should still primarily reflect the efficiency of synNotch cleavage under different conditions. (PDF 1657 kb) [file 12915_2019_625_MOESM1_ESM.pdf]

A

anti-NOTCH

Apical

Basal

control

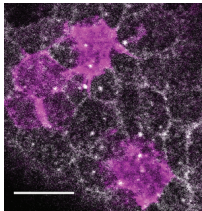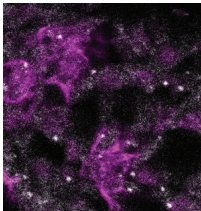

sqhAA

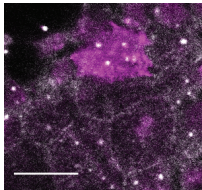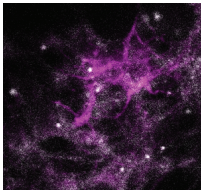

B

anti-DELTA

Apical

Basal

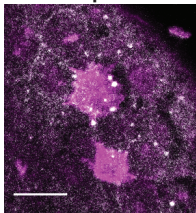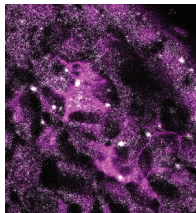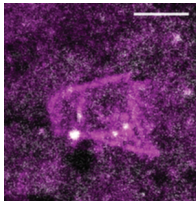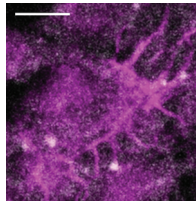

Supplement: Supplementary file 5 — Figure S2. Localization of Notch and Delta with decreased Myosin II activity. In tissues expressing control (LifeActRuby) or sqhAA constructs in SOP cells (tubGAL80ts; neur-GAL4, UAS-GMCA) we observe no differences in (A) Notch localization or (B) Delta localization at a single apical section and basal projection (over 2 μm). Scale bars, 10 μm and 5 μm (sqhAA anti-Delta panels). (PDF 4856 kb) [file 12915_2019_625_MOESM2_ESM.pdf]

A

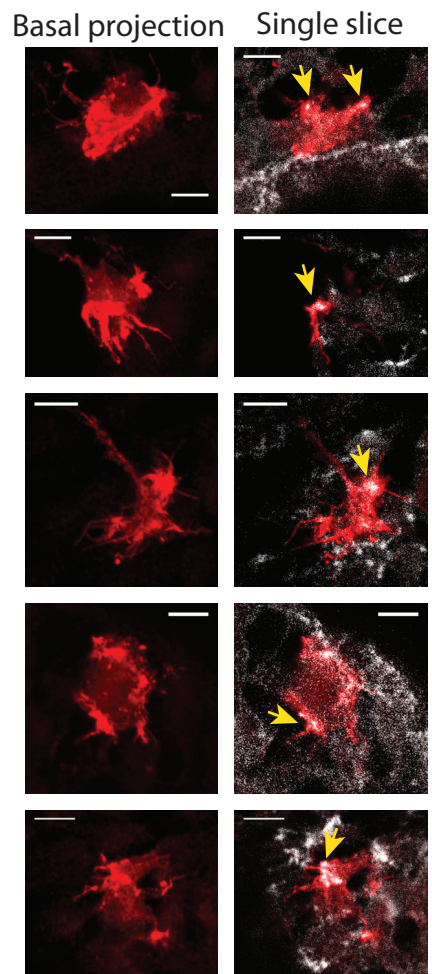

B

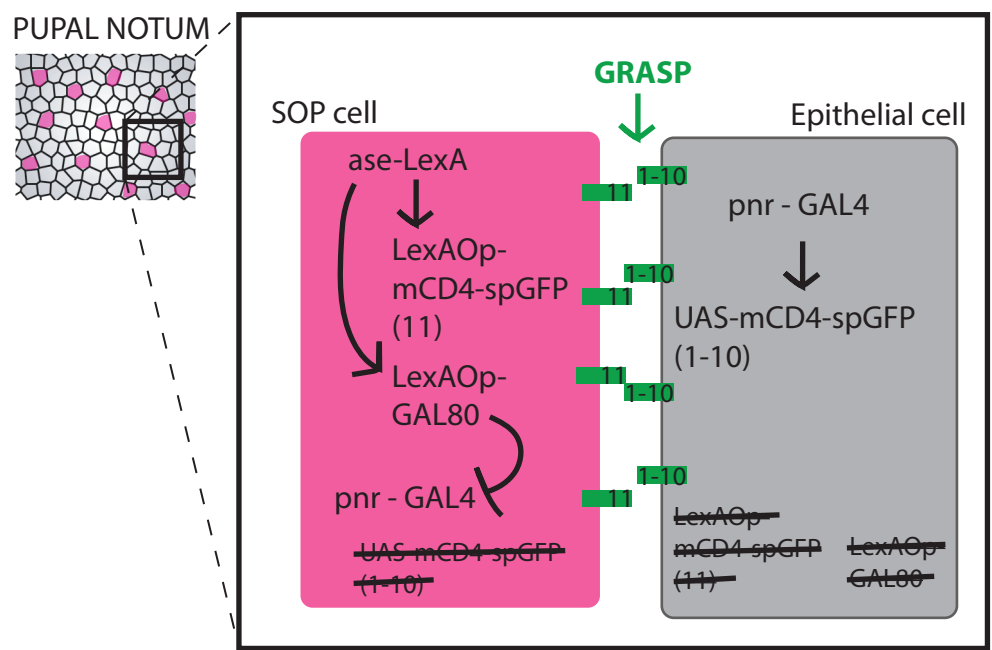

Supplement: Supplementary file 7 — Figure S4. Data supporting main Fig. 6. (A) additional panels of the same genotype and treatment as in Fig. 6B-B”. Filamentous actin in red, phosphorylated myosin regulatory light chain in greyscale. Basal projection images are a maximum intensity projection over 2–4 μm to visualize protrusions. Single slice images are a single z-plane with in the projection images that show the pMRLC puncta. (B) Cartoon to clarify the genetics of the GRASP experiment in Fig. 6C-C”. (PDF 3497 kb) [file 12915_2019_625_MOESM6_ESM.pdf]
