# Supplementary material for: A role for actomyosin contractility in Notch signaling
Source: BMC Biol. 2019 Feb 11;17:12. doi: 10.1186/s12915-019-0625-9 (PMC6369551; doi:10.1186/s12915-019-0625-9)
Supplement: Supplementary file 6 — Figure S3. Role of ligand endocytosis in Notch signaling. (A) Delta ligand endocytosis assay in SOP cells expressing LifeActRuby (control) or zipperDN under tubGAL80ts; neur-GAL4, UAS-GMCA. Endocytosed Delta is specifically visualized through uptake of the anti-Delta (ECD) monoclonal antibody, red puncta (arrows). GFP is stained to visualize the SOP cell body. We find no significant difference in numbers of Delta positive puncta between the control or myosin II perturbed genotypes (graph). (B-C) RNAi against key regulator of ligand endocytosis pathway (liquid facets) lead to a pairing phenotype (indicated by yellow brackets). Pairs of neuralized expressing cells are seen with less frequency in control pupae. (B-C) Scale bars, 25 μm. (D-E) Quantifications of patterns for genotypes in (B-C). (D) Apical ‘cell’ diameter, measuring grouping/pairing. ***p < 0.001 by unpaired t-test. (E) Measurements of the distance between neuralized expressing cells. ***p < 0.001 by unpaired t-test. (n), number of distances measured, N ≥ 3 nota measured for each genotype at ~ 14 h AP. (F) Grouping is observed in cells co-expressing RNAi targeting zipper and liquid facets in SOP cells, compared to either RNAi expressed in SOP cells alone. We note that the grouping phenotype is slightly different in neur-GAL4, UAS-GMCA (D) and tubGAL80ts; neur-GAL4, UAS-GMCA (F) background, for unclear reasons. (PDF 5104 kb) [file 12915_2019_625_MOESM5_ESM.pdf]

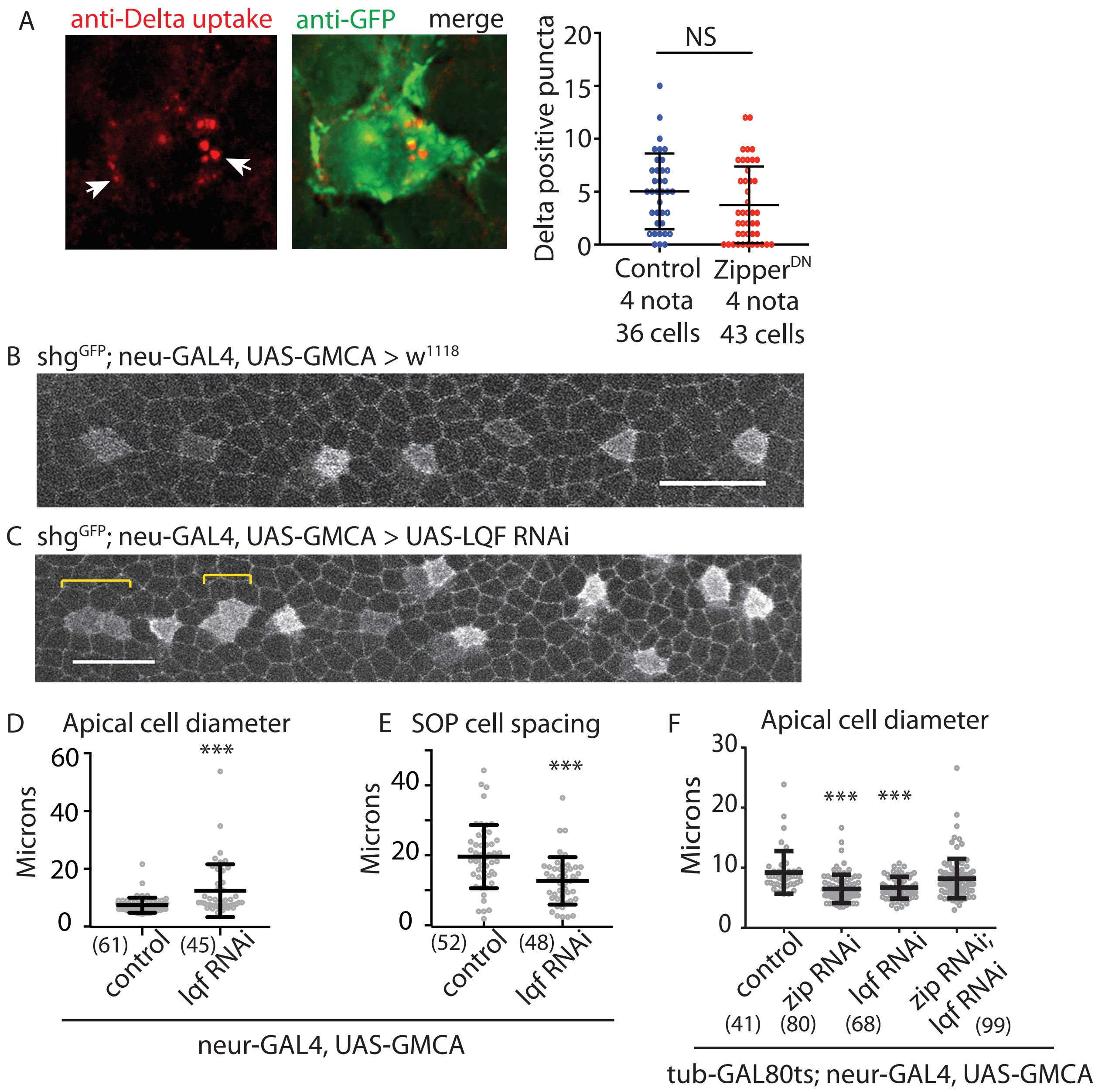
A
anti-Delta uptake
anti-GFP
merge
Delta positive puncta
20
15
10
5
0
NS
Control
4 nota
36 cells
Zipper^{DN}
4 nota
43 cells
B shg^{GFP}; neu-GAL4, UAS-GMCA > w^{1118}
C shg^{GFP}; neu-GAL4, UAS-GMCA > UAS-LQF RNAi
D Apical cell diameter
Microns
60
40
20
0
***
(61) control
(45) lqf RNAi
E SOP cell spacing
Microns
40
20
0
***
(52) control
(48) lqf RNAi
neur-GAL4, UAS-GMCA
F Apical cell diameter
Microns
30
20
10
0
***
***
control (41)
zip RNAi (80)
lqf RNAi (68)
zip RNAi; lqf RNAi (99)
tub-GAL80ts; neur-GAL4, UAS-GMCA
